# Variation in pollinator visitation among garden cultivars of marigold, portulaca, and bidens

**DOI:** 10.1093/jee/toad050

**Published:** 2023-04-28

**Authors:** A Browning, D Smitley, J Studyvin, E S Runkle, Z Y Huang, E Hotchkiss

**Affiliations:** Department of Entomology, Michigan State University, 244 Farm Lane, Room 243, East Lansing, MI 48825-1115, USA; Department of Entomology, Michigan State University, 244 Farm Lane, Room 243, East Lansing, MI 48825-1115, USA; Department of Mathematics and Statistics, University of Wyoming, 1000 E. University Ave. Laramie, WY 82071-303, USA; Department of Horticulture, Michigan State University, 1066 Bogue St, Room A288, East Lansing, MI 48824, USA; Department of Entomology, Michigan State University, 244 Farm Lane, Room 243, East Lansing, MI 48825-1115, USA; Department of Entomology, Michigan State University, 244 Farm Lane, Room 243, East Lansing, MI 48825-1115, USA

**Keywords:** pollinator, annual flower, marigold, bidens, portulaca

## Abstract

Due to declines in pollinator populations, many people are now interested in learning about which annual flowers they can plant in their garden to better support pollinators. However, reports of experimental evaluation of cultivars of annual flowers for attraction to pollinators are scarce. We sampled pollinators visiting six cultivars of marigold (*Tagetes erecta* and *T. patula*), ten cultivars of bidens (*Bidens ferulifolia* and *B. aurea*), and eight cultivars of portulaca (*Portulaca oleracea* and *P. grandiflora*) for two years to compare pollinator visitation rates among cultivars within each flower type. Pollinators collected on flowers in research plots were categorized into four groups, honey bees (*Apis mellifera*), common eastern bumble bees (*Bombus impatiens*), wild bees, and syrphids, to show the proportion of different pollinator visitors to each cultivar. Pollinator visitation rates varied significantly among cultivars of marigold, bidens, and portulaca, with some cultivars having as much as 10-fold the visitation rate of other cultivars of the same flower type. In the second year we also evaluated nectar production and nectar quality of the most and least visited cultivars of portulaca and bidens. Our results show that pollinators have a strong preference for cultivars that produce the most nectar or nectar with the highest sugar content. This research will better inform entomologists, growers, educators, and plant breeders, about which cultivars of marigold, portulaca, and bidens are visited the most by pollinators, and how to accurately determine this at the cultivar level.

Decline in populations of wild bees and honey bees is widespread throughout inhabited areas of the world, with pesticides, habitat loss, disease, and parasites being the leading causes (Potts et al. 2010, Ollerton et al. 2014, Goulson et al. 2015). In 2011, it was estimated that wild bumble bee populations had declined in the United States over a 20-year period, with populations of the four most-impacted species decreasing from 70% to 96% (Cameron et. al 2011). While the number of managed honey bee colonies is increasing in the United States, the proportion of colonies lost quarterly each year still ranges from 9% to 16% (USDA-NASS 2022a). To address these declines, strategies to alleviate pressure on pollinators are being implemented. The Environmental Protection Agency has adopted a pollinator protection initiative, with funds provided by Congress for projects to implement the plan (EPA 2021). An important part of this plan is to increase public awareness of how pollinators are essential for pollination of fruit and vegetables, wildflowers, and landscape ornamentals (Daniels et al. 2020, Theodorou et al. 2020, EPA 2021). In addition, organizations like the Xerces Society and the National Wildlife Federation have increased efforts to provide the public with information on planting pollinator-friendly gardens to support native pollinators (Mader et al. 2011, National Wildlife Federation 2022). A valuable tool for reaching homeowners in the United States about pollinators is through their interest in gardening with annual and perennial flowers. Over 80% of the population in the United States live in urbanized areas (Census Bureau US 2012). In 2021 US homeowners spent $2.38 billion on annual bedding plants (annual flowers) and $1.03 billion on herbaceous perennial ornamental plants (USDA-NASS 2022b). Many garden center customers are now asking how to select flowering plants to support pollinators (Campbell et al. 2017, Wignall et al. 2019). Most published recommendations and nearly all research papers about choosing garden plants for pollinators focus on wildflowers and perennials, with little information about annual flowers (Garbuzov and Ratnieks 2014a). Native and ornamental perennial flowers and woody ornamentals tend to be far more beneficial for pollinators than annual flowers and should be prioritized as food plants for pollinators (Garbuzov et al. 2017, Mach and Potter 2018, Rollings and Goulson 2019). However, since 70% of all herbaceous flowers sold in the United States are annual flowers, it is important to provide information about which ones are most beneficial to pollinators. This is also an opportunity to reach millions of homeowners about the importance of pollinators for agriculture and wildlife. Annual flowers are likely to remain popular because they are bred to bloom continuously throughout the growing season with vibrant colors, while perennials tend to have a shorter bloom cycle and less surface area covered by flowers (Wilde et al. 2015). 

Flower marketers have taken note of customer interest in choosing flowers that support pollinators and have begun placing ‘pollinator-friendly’ labels on plants (Khachatryan et al. 2018, Wollaeger et al. 2015). Surveys show that many people are willing to seek-out and pay more for plants labeled as ‘pollinator-friendly’ (Wignall et al. 2019, Campbell and Steele 2020). However, some plants labeled as ‘pollinator-friendly’ have been shown to have low visitation rates by pollinators (Garbuzov and Ratnieks 2014a, Garbuzov et al. 2017). Due to the lack of research at the cultivar level, and no legal or agreed-upon guidelines, consumers seeking an annual flower that is highly attractive to pollinators may go home with one that is not attractive to pollinators despite it being labeled as ‘pollinator-friendly’.

Previous research on herbaceous perennials provides a framework for the research needed on annual flowers. Perhaps most important is the large variation in pollinator visitation rates that has been observed among cultivars of the same plant type (Erickson et al. 2021). For example, Garbuzov and Rateniks (2014b) found a 10-fold difference in pollinator visitation rates among 13 different cultivars of lavender (*Lavandula* spp.). Similarly, Rollings and Goulson (2019) recorded large differences in visitation rates among cultivars when observing annual and perennial flowers in garden centers. For example, when all cultivars were ranked from 1 to 111 based on the total number of pollinator visits, *Helenium autumnale* ranked #4 while *Helenium* ‘Moorheim beauty’ ranked #24, *Lavandula intermedia* ‘Eidelweis’ ranked #12 while *Lavandula intermedia* ‘Grosso’ ranked #36, and *Lavandula angustifolia* ‘Munstead’ ranked #50 while *Lavandula angustifolia* ‘Little lottie’ ranked # 88.

Although pollinator attraction to the most frequently purchased types of annual flowers has not been experimentally evaluated, a few studies have addressed variation in pollinator visitation among cultivars of a few annuals considered to be attractive to pollinators (Yeargan and Colvin 2009, Erickson et al. 2020). Yeargan and Colvin (2009) focused on butterfly visitation to four cultivars of zinnia (*Zinnia* spp.) and found one cultivar, Lilliput, was visited more often than the other three cultivars. Erickson et al. (2020) evaluated pollinator visitation to five different cultivars each of zinnia, lantana (*Lantana* spp.), sweet alyssum (*Lobularia* spp.), salvia (*Salvia* spp.), and marigold, and found large variation among cultivars of each. This information is helpful, but it also highlights the biggest problem in making recommendations: there may be more than a hundred cultivars of each of the most popular types of annual flowers on the market. For example, marigold, one of the top-ten most purchased annuals (USDA-NASS 2022b), has 181 cultivars listed on the Ball Seed (Chicago, IL) website. With so many cultivars on the market, and little cultivar-specific data available, consumers may want to plant a mix of cultivars and make their own observations to determine which cultivars are most attractive to pollinators.

What is needed most is experimental evaluation of cultivars of selected annual flowers that have been marketed as ‘pollinator friendly’, to inform consumers about the importance of cultivar-level information, and to provide guidelines to plant breeders on how to select for cultivars or lines of annual flowers that are frequently visited by pollinators. In this study we compared pollinator visitation rates among cultivars of three common annual flowers that have been marketed as ‘attractive to pollinators’; marigold, bidens, and portulaca (Table 1). We chose these three types of annual flowers to cover a range in the level of attraction to pollinators. In total, six cultivars of marigold, eight cultivars of portulaca, and ten cultivars of bidens, were evaluated for two years by collecting pollinators on flowers during sample periods. In the second year we also measured nectar volume and sugar concentration produced by flowers of the most and least pollinator-visited cultivars of portulaca and bidens. We hypothesized that cultivars with a greater pollinator visitation rate would produce more nectar, have higher sugar concentrations, or both.

**Table 1.** List of all annual plant cultivars and species used in the experiment

Common name	Species	Cultivar
Marigold	*Tagetes erecta*	Taishan Orange
	*Tagetes erecta*	Antigua Orange
	*Tagetes erecta*	Antigua Primrose
	*Tagetes erecta*	Antigua Yellow
	*Tagetes patula*	Crested Bonanza Yellow
	*Tagetes patula*	Single Disco Marietta
Portulaca	*Portulaca oleracea*	Colorblast Lemon Twist
	*Portulaca oleracea*	Pazzaz Red Flare
	*Portulaca oleracea*	Pazzaz Tangerine
	*Portulaca grandiflora*	Happy Hour Banana
	*Portulaca grandiflora*	Happy Hour Coconut
	*Portulaca grandiflora*	Happy Hour Deep Red
	*Portulaca grandiflora*	Happy Hour Fuchsia
	*Portulaca grandiflora*	Happy Hour Rosita
Bidens	*Bidens ferulifolia*	Bee Dance Painted Red
	*Bidens ferulifolia*	Bee Dance Red Stripe
	*Bidens ferulifolia*	Bee Dance Yellow
	*Bidens ferulifolia*	Bee Happy Orange
	*Bidens ferulifolia*	Bee Happy Red Imperial
	*Bidens ferulifolia*	Bee Bold
	*Bidens ferulifolia*	Blazing Embers
	*Bidens ferulifolia* (Jacq.) DC.	Yellow Splash
	*Bidens ferulifolia* (Jacq.) DC.	Pretty in Pink
	*Bidens aurea* (Aiton) Sherff	Sunbeam

## Materials and Methods

### Plant Preparation

We experimentally evaluated pollinator visitation to six cultivars of marigold, eight cultivars of portulaca, and ten cultivars of bidens (Table 1). Seeds, plugs, and liners were purchased from several commercial greenhouse growers or suppliers, mostly from Raker-Roberta’s Young Plants (Litchfield, MI), Griffin Greenhouse Supplies Inc. (Tewksbury, MA), and Ball Horticultural Co. (West Chicago, IL) for transplanting at the Michigan State University Plant Science Research Greenhouses (East Lansing, MI). Plants were grown by following established floriculture research protocols and culture (e.g., substrate, watering, and fertility; Whitman et al., 2022) starting in February or March, and managed to be grown to a height of 20 to 40 cm, and to be in full bloom by June 1st, when they were planted outside in research plots. Young plants were transferred into 18.9-L plastic pots, with five seedlings or rooted cuttings put into each pot. No pesticides were used. Predatory mites (*Amblyseius cucumeris* and *Hypoaspis miles*) were released prophylactically for biological control of thrips and spider mites, and parasitoids (*Aphidius colemani*, *Aphidius ervi*, and *Aphidius matricariae*) were released for aphid control. One week before the finish date, Osmacote slow-release fertilizer (Marysville, OH) was applied to pots at the labeled rate to provide fertility during the summer season.

**Fig. 1.**
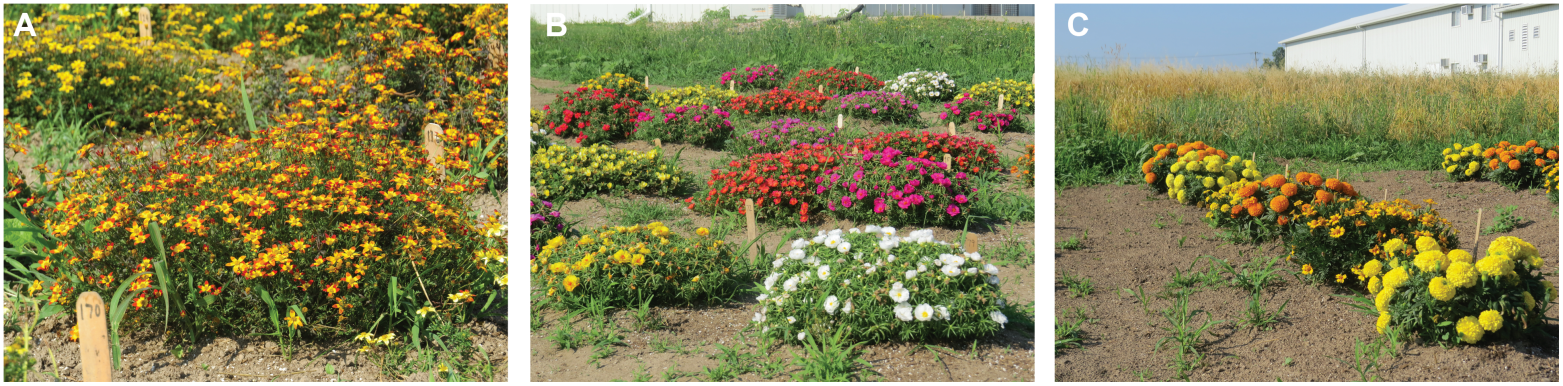
Portulaca (A), bidens (B) and marigold (C) plants in field plots. Each plant was one of six replicate plants of each of eight, ten or six cultivars of portulaca, bidens and marigold, respectively (plot diagram in Fig. 2).

**Fig. 2.**
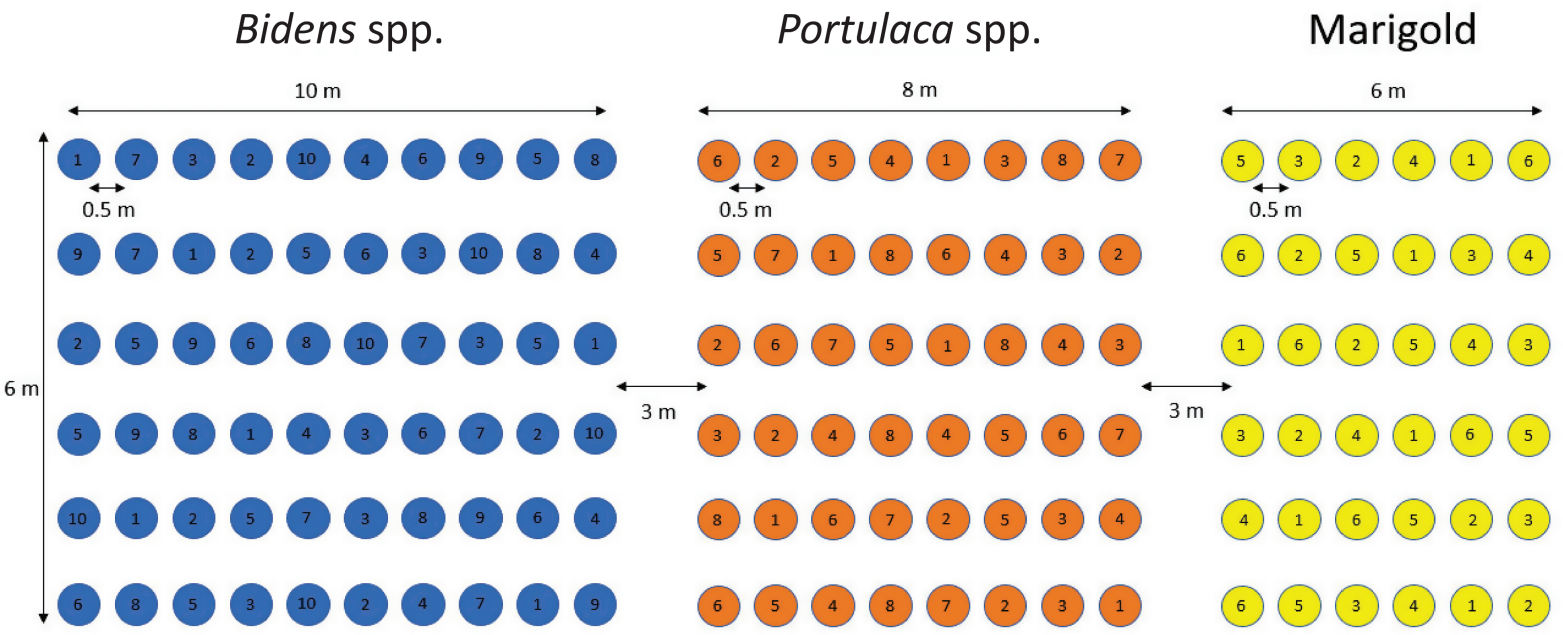
Plot diagram for 2020 and 2021 showing the randomized placement of cultivars in each row and block with the number inside each circle representing a particular cultivar.

On 19 May 2020, a 32 m **×** 10 m area of fallow field, previously used to grow alfalfa, was cleared of any vegetation and rototilled. The surrounding area consisted of alfalfa and a mix of weeds throughout the summer. The plot area was divided into three smaller areas: one for bidens (10 m **×** 6 m), one for portulaca (8 m **×** 6 m), and one for marigold (6 m **×** 6 m). A cleared border of 3 m separated the three plots from each other and from the surrounding field. Containers of plants in each plot area were arranged in a randomized complete block design with six rows. Each row contained one container of each cultivar spaced 0.5 m apart. On June 1st, all test plants in containers were transported to the MSU Pollinator Performance Center, Lansing, Michigan, where they were set next to pre-dug holes according to a plot diagram. The bidens plot consisted of 60 plants (10 cultivars in each row); the portulaca 48 plants (8 cultivars in each row); and the marigold 36 plants (6 cultivars in each row) (Fig. 1 and 2). While planting, potting soil was mixed with the field soil to help the plants with rooting and growth. Ten cm of overhead irrigation was applied each week if there was not adequate rainfall that week (i.e., < 2.5 cm). All weeds were removed by hand hoeing each week. Three hives of *Bombus impatiens* (Cresson) from Biobest Inc. (Leamington, Ontario) were placed 20 m away from the field plots, and two *Apis mellifera* (Linnaeus) hives were placed 15 m from the plots.

On 21 May, 2021, the same field plot area was rototilled in preparation for a second year of research. All of the same cultivars were obtained and grown in the greenhouse as described for 2020. The same experimental design was used, and the surrounding field remained as alfalfa and weeds. Research plants were transported from the greenhouse and planted into field plots on June 1st, 2021, as described for 2020. Two *A. mellifera* colonies were maintained 15 m away from our field plots in 2021, but an additional five colonies were maintained 100 m away. Seventeen colonies of *A. mellifera* were at the Entomology Farm, 6 km away, throughout the summer in 2021.

### Pollinator Collection

The amount of funding available for this research project allowed us to assign one technician, one graduate student, and one undergraduate assistant to collect pollinators from our research plots two half-days per week in June, July, and August of 2020 and 2021. All of the 144 plants were sampled twice per week, for a total of 24 collection periods in 2020, and 20 collection periods in 2021. Pollinators were only collected on days when the temperature was between 15°C and 33°C, and when the wind speed was below 25 kph. Pollinators were also not collected during rain or when rain had occurred earlier in the morning. For each collection period, every plant was observed for one minute. All bees or syrphid flies that were present when the sample period started or any that landed on the flowers during the one-minute period were collected. All specimens were collected with an 18-volt insect vacuum (Bioquip Inc., Rancho Domingo, CA). Collected pollinators were euthanized with ethyl acetate, pinned, labeled, and stored in Cornell University drawers (Bioquip Inc., Rancho Domingo, CA).

Wild bee identifications were made by Jason Gibbs, Curator of the J.B. Wallis and R.E. Roughley Museum of Entomology (Winnipeg, Manitoba, Canada), based on specimens of each morphotype that we sent to him. All collected bees were identified to the species level by comparison to bees identified by Jason Gibbs. This was not possible for half of the individuals in the genera *Lasioglossum* (Halictidae), and *Melissodes* (Apidae), because they were too similar. All syrphid flies were also identified to species, with exception those in the genus *Toxomerus* (Syrphidae). *Bombus impatiens*, *A. mellifera*, and syrphid flies were identified by comparison to voucher specimens collected one or two years earlier in a related research project at the Michigan State University Horticulture Teaching and Research Center, a site that is located 1.5 km away. Syrphid flies were identified to species using the Field Guide to the Flower Flies of Northeastern North America (Skevington et al. 2019). All identifications, other than for wild bees, were confirmed by Gary Parsons, Emeritus Curator of the Michigan State University Arthropod Research Collection. Voucher specimens for all species collected for this research were deposited in the MSU Arthropod Research Collection.

### Nectar Collection

In 2021, nectar was collected from the most and least visited cultivar of bidens (Pretty in Pink and Bee Happy Red Imperial, respectively) and portulaca (Pazzaz Tangerine and Happy Hour Rosita, respectively), based on the mean pollinator visitation per cultivar the previous year. Four plants from each of the four cultivars (16 plants) were selected for nectar sampling. However, we do not have nectar data for the most and least visited cultivars of marigold. Although marigold flowers produce nectar that bees can access, we were unable to extract and measure the amount present because the thin layer of nectar adheres to the sides of densely packed florets and petals. Nectar was sampled from the most and least visited cultivars of bidens and portulaca by placing four pollinator exclusion bags on each of the 16 plants at 09:00 HR one day before the nectar sampling day. Each exclusion bag covered three flowers. On the sampling day, nectar was collected at one-hour intervals from 09:00 to 16:00 HR. Two replicate plants of each cultivar were sampled on 15 Aug. and two replicated plants on 16 Aug. Weather conditions were sunny or partly cloudy with no rain. Nectar from both portulaca cultivars was collected from bagged flowers with 1.0 µl capillary tubes. One sample consisted of all the nectar that could be extracted from a single flower. Five nectar samples were collected from each of ‘Pazzaz Tangerine’ plants and five samples from ‘Happy Hour Rosita’ plants. Individual bidens flowers did not produce enough nectar for a reliable sample because they are a composite flower with multiple florets within one inflorescence. For that reason, nectar samples were taken from five florets per head and combined into one floral head sample. Five floral head nectar samples were taken from each ‘Pretty in Pink’ plant for a total of 20 samples. We were unable to collect enough nectar using the capillary method from the least-visited cultivar, ‘Bee Happy Red Imperial’, due to the small amount of nectar produced. The volume of nectar for each sample collected by the capillary method was determined by using a digital caliper (AdoricLife, Orlando, FL) to measure how much of the 1.0 µl capillary tube was filled. The length of the filled capillary tube was then converted into microliters. Sugar concentrations for each sample were determined by using a Reichert Inc. (Depew, NY) Brix50 refractometer.

Due to the small amount of nectar produced by ‘Bee Happy Red Imperial’ flowers, nectar was sampled using centrifugation. The centrifugation method was standardized with the capillary tube method, by using both methods to sample ‘Pretty in Pink’, a cultivar that produced enough nectar to sample with capillary tubes (Table 3). For each cultivar, mature floral heads were removed from the bagged flowers and placed individually into 500 µl PCR tubes (with florets facing downward) after all petals were removed. The PCR tubes were then placed into an Eppendorf (Hamburg, Germany) 5415D centrifuge that was run for two minutes at 3000G to extract the nectar. The nectar was collected from the bottom of the PCR tube with a 1.0 µl capillary tube. One sample consisted of the nectar obtained from one floral head. Five nectar samples were measured for ‘Pretty in Pink’ and 14 samples were measured for ‘Bee Happy Red Imperial’. The volume of nectar in each sample was determined using the same method as above. Sugar concentrations were also determined for each sample as previously described.

**Table 2.** List of all pollinators collected with identification of each to genus or species, and number of each collected

Pollinator group	Family	Genus	Species	Collected (*n*)
*Apis mellifera* (*n* = 551)	Apidae	*Apis*	*mellifera*	551
*Bombus impatiens* (*n* = 49)	Apidae	*Bombus*	*impatiens*	49
Wild Bees^a^ (*n* = 920)	Andrenidae	*Calliopsis*	*andreniformis*	7
	Apidae	*Melissodes*	*bimaculatus*	4
		*Melissodes*	*sp.*	3
		*Xylocopa*	*virginica*	3
	Halictidae	*Agapostemon*	*sericeus*	6
		*Agapostemon*	*texanus*	1
		*Agapostemon*	*virescens*	19
		*Augochlorella*	*aurata*	12
		*Halictus*	*confusus*	130
		*Halictus*	*ligatus*	247
		*Halictus*	*rubicundus*	12
		*Lasioglossum*	*admirandum*	2
		*Lasioglossum*	*coriaceum*	2
		*Lasioglossum*	*ellisiae*	6
		*Lasioglossum*	*ephialtum*	18
		*Lasioglossum*	*hitchensi*	2
		*Lasioglossum*	*imitatum*	1
		*Lasioglossum*	*leucocomus*	3
		*Lasioglossum*	*leucozonium*	3
		*Lasioglossum*	*paradmirandum*	6
		*Lasioglossum*	*pectorale*	1
		*Lasioglossum*	*pilosum*	169
		*Lasioglossum*	*weemsi*	4
		*Lasioglossum*	*sp.*	257
		*Sphecodes*	*mandibularis*	1
	Megachilidae	*Anthidium*	*oblongatum*	1
Syrphid (*n* = 1,038)	Syrphidae	*Allograpta*	*obliqua*	9
		*Eristalis*	*flavipes*	2
		*Eristalis*	*arbustroum*	7
		*Eristalis*	*obscura*	7
		*Eristalis*	*tenax*	178
		*Helophilus*	*fasciatus*	23
		*Helophilus*	*latifrons*	22
		*Sphaerophoria*	*scripta*	22
		*Syritta*	*pipiens*	14
		*Syrphus*	*ribesii*	15
		*Toxomerus*	*sp.*	735
		*Tropidia*	*quadrata*	4

^a^refers to all bees other than *Apis mellifera* and *Bombus impatiens*.

**Table 3.** Comparison of nectar volume or sugar concentration produced by the most pollinator-visited (Pazzaz Tangerine) and least visited (Happy Hour Rosita) cultivars of portulaca, and the most visited (Pretty in Pink) and least visited (Bee Happy Red Imperial) cultivars of bidens. The capillary method worked well for both cultivars of portulaca, but centrifugation was necessary to collect enough nectar to measure the amount produced by the bidens cultivar, Bee Happy Red Imperial. Means are compared with an unpaired Wilcoxon test

Species and cultivar	Year	Mean pollinator visitation rate (visits per min)	Nectar volume by capillary method in 2021 (µl)	Nectar volume by centrifuge method in 2021 (µl)	Sugar concentration by capillary method in 2021 (%)	Sugar concentration by centrifuge method in 2021 (%)
*Portulaca oleracea* *‘*Pazzaz Tangerine’	2020	1.42 ± 0.22	0.27 ± 0.13	NA	10 ± 6	NA
	2021	1.24 ± 0.2				
*Portulaca grandiflora* *‘*Happy Hour Rosita’	2020	0.19 ± 0.07	0.04 ± 0.07	NA	2 ± 2	NA
	2021	0.48 ± 0.12				
W and P values for comparison of *‘*Pazzaz Tangerine’ with *‘*Happy Hour Rosita’ nectar means			W = 421 *P* < 0.0001		W = 372 *P* < 0.001	
*Bidens ferulifolia* ‘Pretty in Pink’	2020	1.17 ± 0.17	0.1 ± 0.05	0.08 ± 0.05	5 ± 2	3 ± 2
	2021	2.47 ± 0.28				
*Bidens ferulifolia* *‘*Bee Happy Red Imperial’	2020	0.1 ± 0.05	NA	0.01 ± 0.02	NA	0.1 ± 0.1
	2021	0.17 ± 0.07				
W and P values for comparison of ‘Pretty in Pink’ with *‘*Bee Happy Red Imperial’				W = 53 *P* < 0.01		W = 56 *P* < 0.001

The capillary method worked well for both cultivars of portulaca, but centrifugation was necessary to collect enough nectar to measure the amount produced by the bidens cultivar, Bee Happy Red Imperial. Means are compared with an unpaired Wilcoxon test.

### Data Analysis

All analyses were conducted in R Version 4.1.2 (R Core Team 2021). Pollinators collected from research plots were split into four pollinator groups: *A. mellifera*, *B. impatiens*, Wild Bees, and Syrphids. Although mean visitation rates for each group of pollinators to each cultivar are indicated in bar graphs with the bars divided into a different color for each pollinator group, the statistical comparison of mean visitation rates among cultivars of marigold, portulaca, and bidens were made after combining the four groups of pollinators into an overall pollinator visitation rate. One-minute sampling periods to each of the six replicate cultivars were summed for all collection periods each year to determine a mean visitation rate for each annual. The results are presented as overall pollinator visits per minute for each cultivar in each year. Comparisons were made among cultivars of marigold, portulaca, and bidens within each year, but no comparisons are made among the three different flower types. A Poisson regression approach was used to assess the differences in pollinator visitation rates among the cultivars of each annual (Bilder and Loughin 2014). All pairwise tests between the cultivars were done within the Poisson regression framework. To correct for the compounding of the alpha level, a Tukey was utilized (Kuehl 2000). This analysis was done for each year separately, with pollinator visitation counts set as the response variable and the time of sampling as the dependent variable. Marigold, portulaca, and bidens were analyzed separately to compare visitation rates among the cultivars of each.

Nectar volume and nectar sugar concentration for the cultivar of bidens with most pollinator visits was compared to that for the least visited cultivar using an unpaired Wilcoxon test. The same approach was used to compare the most and least visited cultivars of portulaca.

## Results

In 2020 1,290 pollinators were collected from flowers, and in 2021 1,268 were collected, for a total of 2,558 for both years combined (Table 2). In 2021, more honey bees were observed in our research plots than in 2020. This may be due to the five additional honey bee colonies maintained within 100 m of our research plots in 2021. In contrast, more wild bees were observed in 2020 than in 2021. Marigold and portulaca cultivars with the most honeybee visits in 2021 tended to have fewer wild bee visits in 2021 compared with 2020. This is consistent with previous observations of wild bee suppression when honey bee activity increases (Thomson 2004, Wojcik et al. 2018). Syrphids visited flowers at a similar observed rate in 2020 and 2021. Differences in pollinator activity from 2020 to 2021 did not affect comparisons of pollinator visits among cultivars because the comparisons were made within each year.

**Fig. 3.**
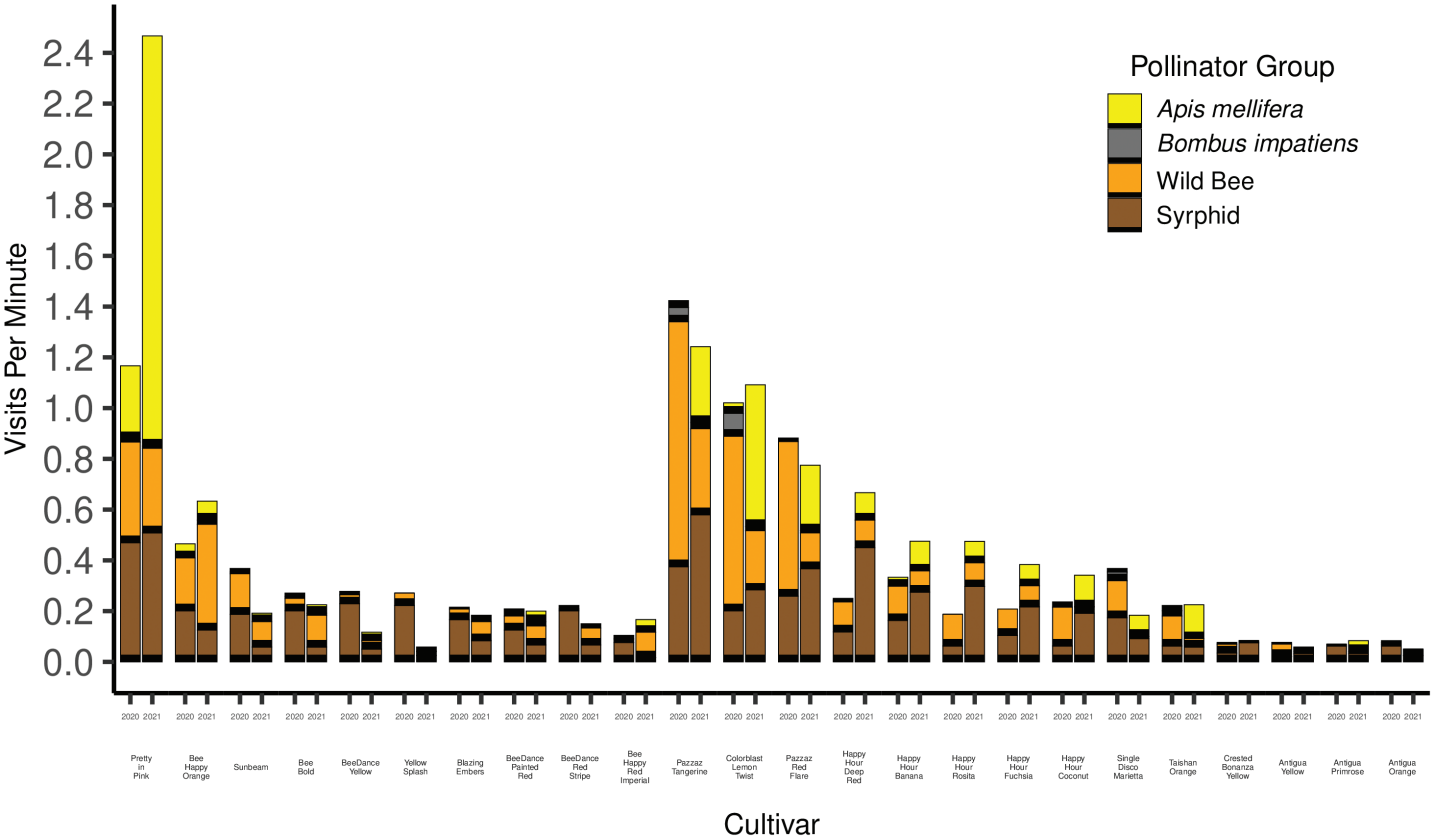
Combined pollinator visits per minute for all cultivars of portulaca (a – h), bidens (a – j) and marigold (a – f). For each pair of bars, the bar on the left represents visits per minute in 2020 and the bar on the right for 2021. Cultivar names and larger figures for comparison of cultivars of portulaca, bidens and marigold appear in Figs. 4–6.

Syrphid flies were the most abundant pollinator, with 1,038 individuals collected from 12 species in 8 genera. Wild bees were next, with 920 individuals in four families, six genera, and 20 species. Of the wild bees, species of Halictidae were by far the most abundant, accounting for 902 of the 1,038 wild bees collected. All collected species of *Lasioglossum* were identified by Jason Gibbs based on a large subsample of morphotypes, but some were so similar that we were unable to identify them to species after comparison to specimens returned to us by Gibbs. This resulted in 257 individuals, about half of all *Lasioglossum* that were collected, to be listed as *Lasioglossum* sp. A total of 551 honey bees (*A. mellifera*) and 49 bumble bees were collected, with all bumble bees being *B. impatiens*. Not more than 20 individuals of any other type of pollinator were collected and were therefore excluded from our data analysis. The proportions of syrphid flies, wild bees, honey bees, and bumble bees were similar in 2020 and 2021. The most pollinator visits to marigold and bidens flowers were by syrphid flies (43–46%), followed by *A. mellifera* (26–27%), and wild bees (26%, Fig. 3). Visitation to portulaca flowers followed a different pattern, with wild bees making the most visits (40.5%), followed by syrphid flies (40%), and *A. mellifera* (17.5%, Fig. 3).

Although we observed a trend of a 3-fold and 5-fold greater visitation rate to bidens and portulaca flowers compared with marigold flowers, our experimental design only allows us to compare pollinator visitation rates among cultivars of marigold, among cultivars of bidens, and among cultivars of portulaca. Greater visitation to portulaca and bidens flowers by all pollinators combined (0.63 visits/min and 0.39 visits/min, respectively) allowed for better separation of visitation rates to cultivars than for marigold flowers (0.13 pollinators/min).

**Fig. 4.**
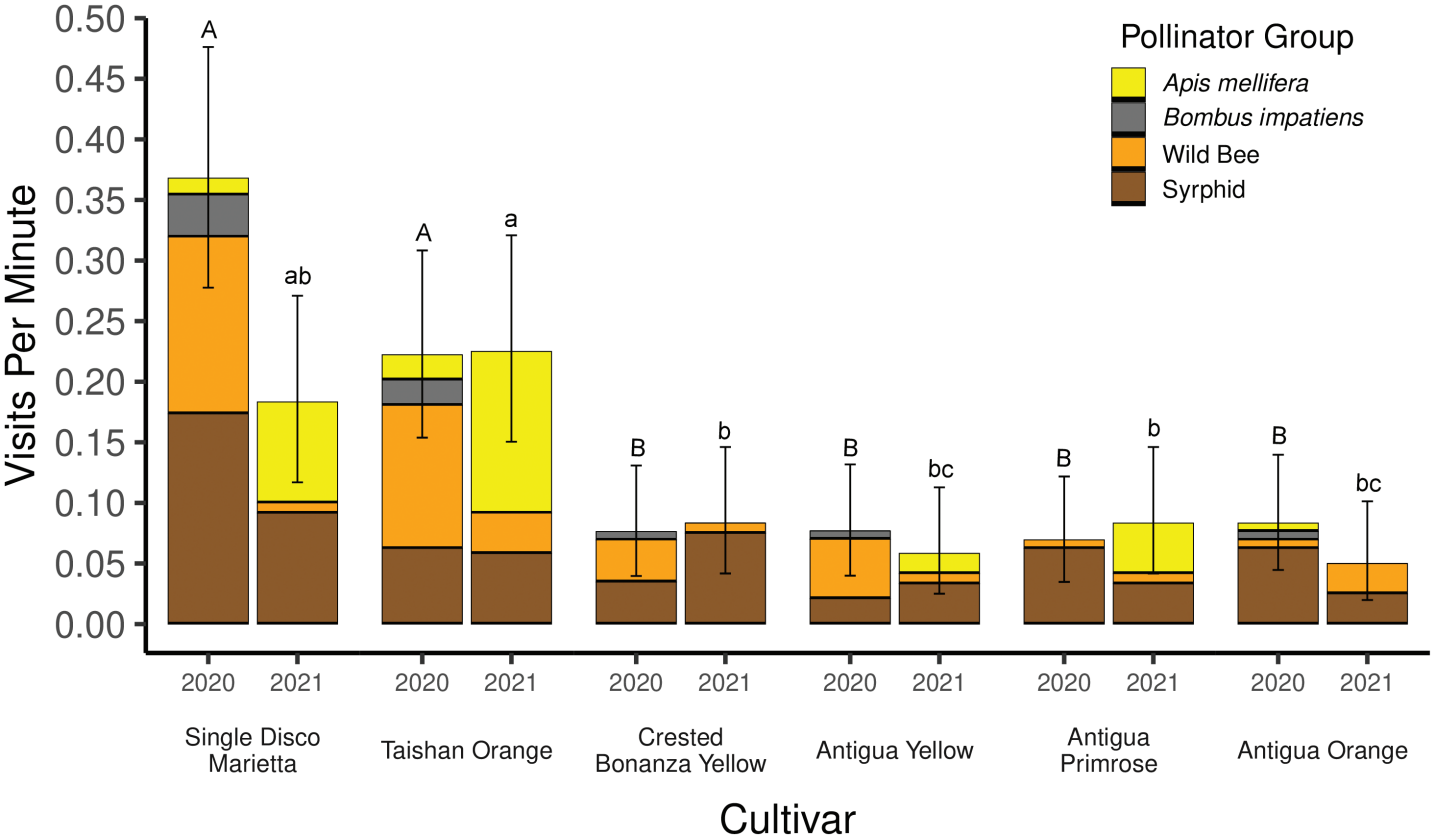
Mean combined pollinator visitation rate to six cultivars of *Marigold* (marigold) in 2020 and 2021. Error bars are 95% confidence intervals. Cultivar means are compared within 2020 (capital letters above bars) and 2021 (lower case letters above bars). Bars with the same capital letter or the same lower-case letter are not significantly different (P = 0.05).

**Fig. 5.**
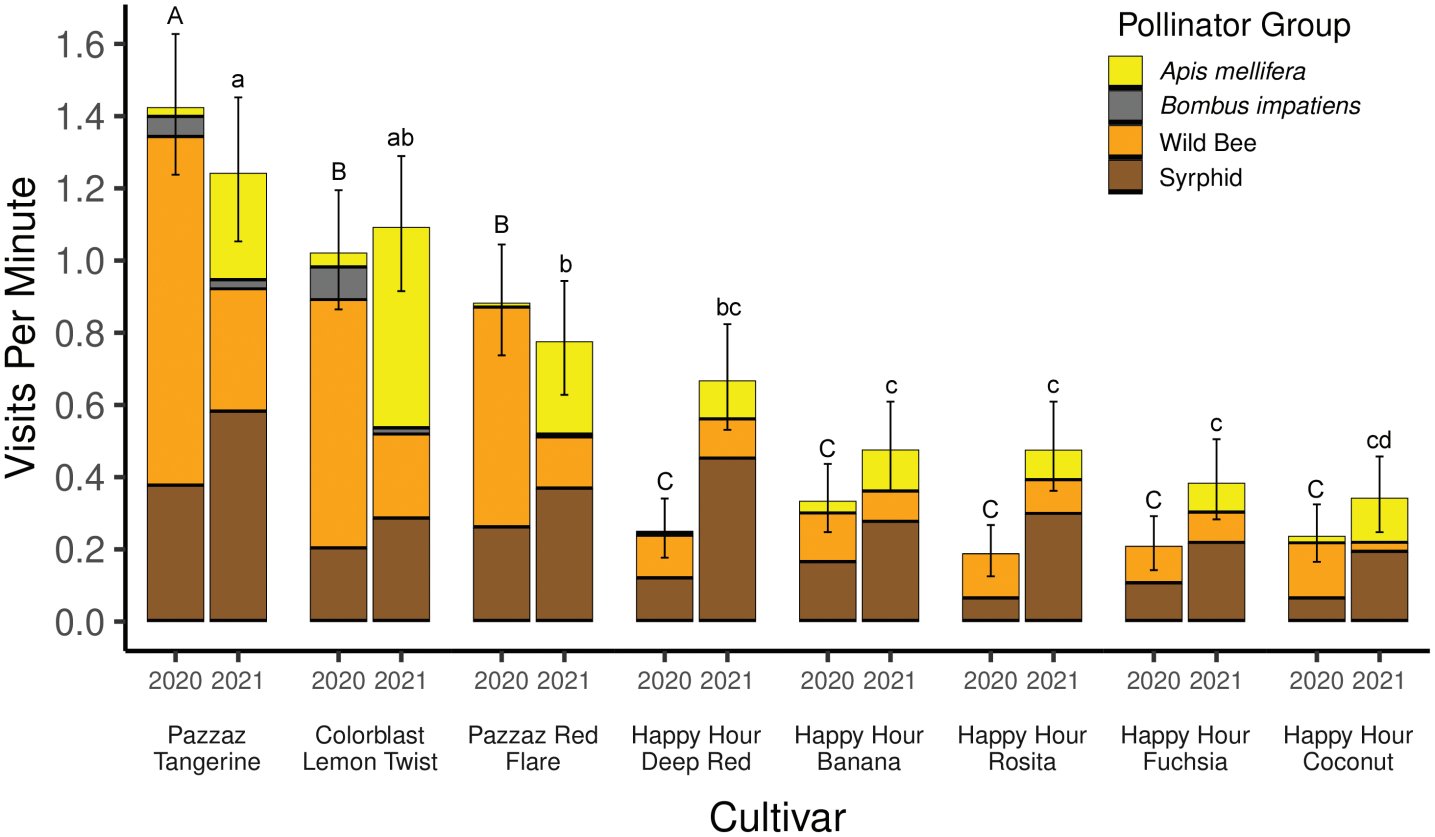
Mean combined pollinator visitation rate to eight cultivars of portulaca in 2020 and 2021. Error bars are 95% confidence intervals. Cultivar means are compared within 2020 (capital letters above bars) and 2021 (lower case letters above bars). Bars with the same capital letter or the same lower-case letter are not significantly different (P = 0.05).

**Fig. 6.**
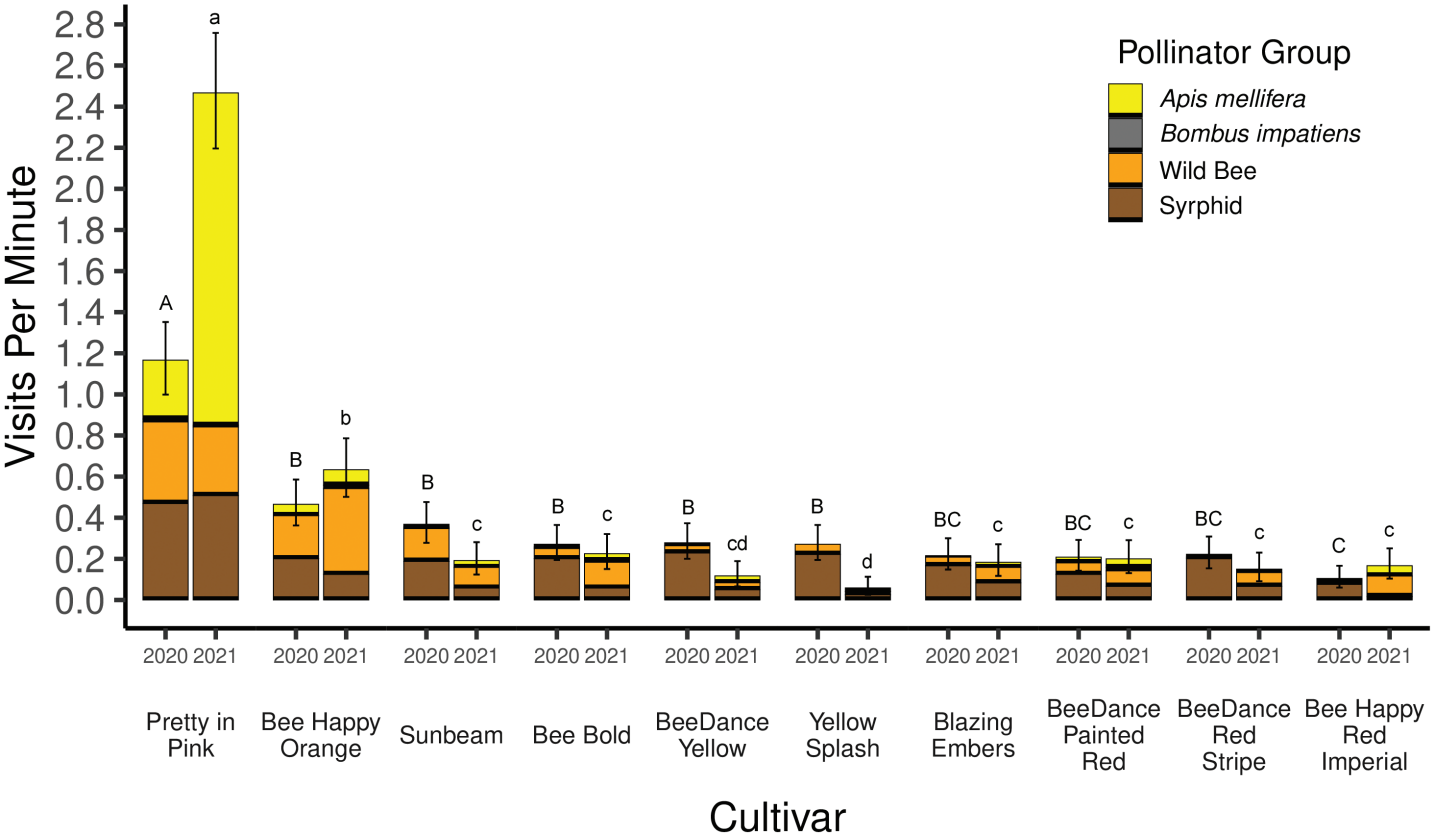
Mean pollinator visitation rate to ten cultivars of bidens in 2020 and 2021. Error bars are 95% confidence intervals. Cultivar means are compared within 2020 (capital letters above bars) and 2021 (lower case letters above bars). Bars with the same capital letter or the same lower-case letter are not significantly different (P = 0.05).

Pollinator visitation rates among cultivars varied widely within each plant type. In the same year, visitation among cultivars of marigold varied as much as 5-fold, while bidens cultivars varied as much as 24-fold, and portulaca cultivars, 7-fold (Figs. 4–6). The volume of nectar and the concentration of sugar in nectar collected from flowers of the most-visited cultivars were greater than that for the least-visited cultivars of portulaca and bidens (Table 3). Marigold flowers did not produce enough nectar to be measured. In the following three sections, results demonstrating large variation among cultivars in pollinator visitation rates as well as nectar production and sugar concentrations, are presented separately for marigold, bidens, and portulaca.

### Marigold

Almost half of the pollinators collected on marigold flowers were syrphids (43.4%, Fig. 4), followed by *A. mellifera* (26.8%), wild bees (25.9%), and *B. impatiens* (3.9%). The pollinator visitation rate to ‘Taishan Orange’ flowers in 2020 (0.22 ± 0.08) and 2021 (0.23 ± 0.08), and to ‘Single Disco Marietta’ flowers in 2020 (0.37 ± 0.1), were greater than the visitation rates to the four remaining cultivars of marigold, which all had visitation rates less than 0.10 visits per minute. There were no differences among the four least-visited cultivars, ‘Crested Bonanza Yellow’, ‘Antigua Yellow’, ‘Antigua Primrose’, and ‘Antigua Orange’, in 2020 or 2021 (Fig. 4).

### Bidens

Syrphids were the most abundant visitors to bidens flowers (46.0%), followed by *A. mellifera* (26.0%), wild bees (26.0%), and *B. impatiens* (2.0%) (Fig. 6). ‘Pretty in Pink’ had the greatest mean visitation rate in 2020 (1.17 ± 0.17) and in 2021 (2.47 ± 0.28), significantly different (*P* < 0.05) from all other cultivars (Fig. 5). In both years, ‘Bee Happy Orange’ had significantly greater mean pollinator visits per minute in 2020 (0.47 ± 0.11) and 2021 (0.63 ± 0.14) than ‘Blazing Embers’, ‘BeeDance Painted Red’, ‘BeeDance Red Stripe’, and ‘Bee Happy Red Imperial’ (Fig. 5). In 2021 ‘Bee Happy Orange’ also had significantly more pollinator visits than ‘Sunbeam’ (0.19 ± 0.08), ‘BeeDance Yellow’ (0.12 ± 0.06), and ‘Yellow Splash’ (0.06 ± 0.04) (Fig. 5). The volume of nectar collected from the cultivar of bidens with the greatest mean pollinator visits per minute in 2020, ‘Pretty in Pink’, was significantly greater than the volume of nectar collected from the least visited cultivar, ‘Bee Happy Red Imperial’, using the centrifuge method (*W* = 53, *P* < 0.01) (Table 3). Similarly, the nectar sugar concentration from the flowers of ‘Pretty in Pink’ was significantly greater than that from ‘Bee Happy Red Imperial’, also using the centrifuge method (*W* = 56, *P* < 0.001).

### Portulaca

Most of the pollinators collected on portulaca flowers were wild bees (40.5%) and syrphid flies (40.2%), followed by *A. mellifera* (17.5%) and *B. impatiens* (1.8%, Fig. 5). The most preferred cultivars to all pollinators combined were ‘Pazzaz Tangerine’ and ‘Colorblast Lemon Twist’, which had visitation rates of 1.42 ± 0.22 and 1.02 ± 0.16, respectively in 2020 and 1.24 ± 0.2 and 1.09 ± 0.19, respectively, in 2021. Visits to these two cultivars were significantly greater than visits to any other cultivar, with the exception of ‘Pazzaz Red Flare’ which had similar visitation rates in 2020 (0.88 ± 0.15) and in 2021 (0.77 ± 0.16). ‘Pazzaz Red Flare’ also had significantly greater visitation rates in both years than all remaining cultivars with the exception of ‘Happy Hour Deep Red’ in 2021 (0.67 ± 0.14). There were no differences among the five least-visited cultivars in 2020 or 2021.‘Happy Hour Deep Red’ and ‘Happy Hour Rosita’ had significantly higher mean pollinator visitation rates in 2021 (0.67 ± 0.14 and 0.48 ± 0.12, respectively) than in 2020 (0.21 ± 0.07 and 0.19 ± 0.07, respectively). All other cultivars did not vary from year to year in pollinator visits per minute.

The volume of nectar collected from the cultivar of portulaca with the greatest mean pollinator visits per minute in 2020, ‘Pazzaz Tangerine’, was significantly greater than that collected from the least visited cultivar, ‘Happy Hour Rosita’ (*W* = 421, *P* < 0.0001) (Table 3). Similarly, the sugar concentration of nectar from ‘Pazzaz Tangerine’ flowers was significantly greater than that from ‘Happy Hour Rosita’ flowers (*W* = 372, *P* < 0.001).

## Discussion

Our results demonstrate that pollinator visitation to marigold, portulaca, and bidens, all of which have been promoted as being attractive to pollinators, varies greatly among cultivars of each (Figs. 4–6). Therefore, any one of these types of flowers cannot be accurately described as more ‘attractive to pollinators’ or more ‘pollinator-friendly’ than either of the other two flower types without cultivar-specific information. Even marigold, the least visited of the three flower types, had two cultivars that were visited nearly as often as seven cultivars of bidens and four cultivars of portulaca (Fig. 3). In a similar field experiment, Erickson et al. (2020) evaluated five cultivars, each, of five types of annual flowers, available at garden centers (lantana, sweet alyssum, pentas, marigold, and zinnia) for visitation by pollinators. They also found large variation among cultivars of sweet alyssum (15-fold), pentas (5-fold), and zinnia (5-fold), but much less variation among cultivars of lantana (1.7-fold) and marigold (1.5-fold). Our results indicated much more variation among cultivars of marigold (7-fold) than Erickson et al. (2020), but only one cultivar, ‘Taishan Orange’, appeared in both studies. Furthermore, three cultivars in our study were from the same breeding line: ‘Antigua Yellow’, ‘Antigua Primrose’, and ‘Antigua Orange’, and four of the five cultivars evaluated by Erickson et al (2020) were from a different breeding line: ‘Alumia Deep Orange’, ‘Alumia Flame’, ‘Alumia Red’, and ‘Alumia Yellow’. The consistency in results among marigold cultivars in the same breeding line by Erickson et al. (2020) and in our study, suggests that there may be more variation among cultivars from different breeding lines than cultivars in the same breeding. However, Erickson et al. (2020) reported as much as a 5-fold difference among ‘Zahara Red’ and the other three cultivars of zinnia in the same breeding line. With so many cultivars of annual flowers being sold at garden centers, it may be more efficient to begin evaluating cultivars by choosing one cultivar from as many different breeding lines as possible.

Our purpose was to compare pollinator visitation among cultivars of portulaca, bidens, and marigold, and not to determine why there are differences. However, we did measure one factor known to be important: nectar production (Silva and Dean 2000, Somme et al. 2015, Mallinger and Prasifka 2017, Van Rijn and Wäckers 2016). Our results suggest that the volume and sugar content of nectar in portulaca and bidens flowers is important to pollinators. The most attractive cultivars produced at least 10-fold more nectar by volume, and the sugar concentration was at least 10-fold greater than that of the least attractive cultivars (Table 3). Although nectar volume and sugar content are important, many other factors influence the visitation rates of pollinators. We did not measure or attempt to account for several potentially confounding factors: floral surface area, competition among honey bees and wild bees, or floral resources in surrounding areas (Goulson 2003). 

In the future, evaluating nectar production and sugar concentration may be one way that plant breeders could collect data to support labeling of cultivars as ‘pollinator-friendly’. Evaluating nectar production would also help when attempting to compare results from different field sites, because there may be large differences in pollinator visitation rates to the same cultivars when they are grown at different field sites due to the relative abundance of pollinators in and around the research plots. Using a ‘standard’ cultivar as one treatment at different research sites, can also be used to help compare results from one site to another.

Although our experimental design does not allow comparison of pollinator visits to a particular cultivar among years, future experimenters may want to do so because one cultivar of marigold, ‘Single Disco Marietta’ had twice as many visits in 2020 compared with 2021, while all other cultivars had a similar rate of visitation in both years (Fig. 4). Similarly, pollinator visitation to two cultivars of portulaca (‘Happy Hour Deep Red’ and ‘Happy Hour Rosita’) and two cultivars of bidens (‘Pretty in Pink’ and ‘Yellow Splash’) were different in 2020 and 2021 (Fig. 6). A significant increase or decrease in the pollinator visitation rate from one year to the next for an annual flower cultivar could indicate that the amount or quality of floral resources available to pollinators has changed. One explanation for this which could be explored in future research is that nectar production by flowers of a particular cultivars may vary from year to year due to the selection process used by plant breeders. Cultivars of annual flowers are selected each year based on vigor, growth habit, pest resistance, color, and floral display; with traits like nectar and pollen production being ignored (Comba et al. 1999, Horn 2002, Guo and Warner 2020). Collecting nectar as part of the selection process for ‘pollinator friendly’ cultivars could avoid this problem.

Syrphid flies were collected from every cultivar of marigold, portulaca, and bidens during both years of pollinator collections, and along with wild bees, were the most abundant category of pollinators (Figs. 4–6). Attention to syrphid flies as a functional pollinator group, as well as a form of biological control, has increased in recent years (Klecka et al. 2018, Doyle et al. 2020, Dunn et al. 2020). Because the larvae of syrphids are predators of aphids and psyllids, which are common garden pests, they are valuable for biological control, which is important when avoiding pesticides to protect pollinators (White et al 1995, Irvin et al. 2021).

In recent years people have become more interested in learning what they can do to help pollinators, and many are choosing flowers from garden centers that are labeled as ‘pollinator friendly’. Our results which identify cultivars of marigold, portulaca, and bidens that are most attractive to pollinators, along with previous work by Erickson et al. (2020) on cultivars of zinnia, sweet alyssum, marigold, lantana, and pentas, will allow educators to recommend cultivars that are often 10 to 20-fold more visited by pollinators than other cultivars of the same flower types. This will help support pollinators in urban landscaping where vibrant colors that last all season are preferred for hanging baskets and seasonal beds. This research will also inform plant breeders on how to evaluate and ultimately develop breeding lines that are heavily visited by pollinators, and can therefore be correctly marketed that way.

## References

[CIT0001] Bilder CR , Loughin TM. Analysis of categorical data with R. Boca Raton, Florida: CRC Press; 2014.

[CIT0002] Cameron SA , Lozier JD, Strange JP, Koch JB, Cordes N, Solter LF, Griswold TL. Patterns of widespread decline in North American bumble bees. PNAS. 2011:108:662–667. 21199943 10.1073/pnas.1014743108 PMC3021065

[CIT0003] Campbell B , Khachatryan H, Rihn A. Pollinator-friendly plants: reasons for and barriers to purchase. HortTechnology. 2017:27(6):831–839. 10.21273/horttech03829-17

[CIT0004] Campbell B , Steel W. Impact of information type and source on pollinator-friendly plant purchasing. HortTechnology. 2020:30:122–128.

[CIT0005] Census Bureau US. Growth in urban population outpaces rest of nation, census bureau reports. Suitland, MD: U.S. Census Bureau; 2012.

[CIT0006] Comba L , Corbet SA, Barron A, Bird A, Collinge S, Miyazaki N, Powell M. Garden flowers: insect visits and the floral reward of horticulturally-modified variants. Ann Bot (Lond). 1999:83:73–86.

[CIT0007] Daniels B , Jedamski J, Ottermanns R, Ross-Nickoll M. A ‘plan bee’ for cities: Pollinator diversity and plant-pollinator interactions in urban green spaces. PLoS One. 2020:15(7):e0235492. 10.1371/journal.pone.0235492 32667935 PMC7363068

[CIT0008] Doyle T , Hawkes WLS, Massy R, Powney GD, Menz MHM, Wotton KR. Pollination by hoverflies in the Anthropocene. Proc R Soc B. 2020:287:20200508. 10.1098/rspb.2020.0508 PMC7287354 32429807

[CIT0009] Dunn L , Lequerica M, Reidc CR, Tanya L. Dual ecosystem services of syrphid flies (Diptera: Syrphidae): pollinators and biological control agents. Pest Manag Sci. 2020:76:1973–1979. 32115861 10.1002/ps.5807

[CIT0010] Erickson E , Adam S, Russo L, Wojkik V, Patch HM, Grozinger CM. More than meets the eye? The role of annual ornamental flowers in supporting pollinators. Environ Entomol. 2020:49:178–188. 31755522 10.1093/ee/nvz133

[CIT0011] Erickson E , Patch HM, Grozinger CM. Herbaceous perennial ornamental plants can support complex pollinator communities. Sci Rep. 2021:11(1):17352. 10.1038/s41598-021-95892-w 34462447 PMC8405689

[CIT0012] Garbuzov M , Altonand K, Ratnieks FLW. Most ornamental plants on sale in garden centres are unattractive to flower-visiting insects. PeerJ. 2017:5:e3066. 28286716 10.7717/peerj.3066 PMC5344017

[CIT0013] Garbuzov M , Ratnieks FLW. Listmania: the strengths and weaknesses of lists of garden plants to help pollinators. Bioscience. 2014a:64(11):1019–1026. 10.1093/biosci/biu150

[CIT0014] Garbuzov M , Ratnieks FW. Quantifying variation among garden plants in attractiveness to bees and other ﬂower-visiting insects. Funct Ecol. 2014b:28:364–374.

[CIT0015] Goulson D. Effects of introduced bees on native ecosystems. Annu Rev Ecol Evol Syst. 2003:34(1):1–26. 10.1146/annurev.ecolsys.34.011802.132355

[CIT0016] Goulson D , Nicholls E, Botías C, Rotheray EL. Bee declines driven by combined stress from parasites, pesticides, and lack of flowers. Science. 2015:347:1435. 10.1126/science.1255957 25721506

[CIT0017] Guo Y , Warner RM. Dissecting genetic diversity and genomic background of Petunia cultivars with contrasting growth habits. Hortic Res. 2020:7:155. 10.1038/s41438-020-00373-2 33082962 PMC7528118

[CIT0018] Horn W. 2002. Breeding methods and breeding research. In: Breeding for ornamentals: classic and molecular approaches. Dordrecht, Netherlands: Springer; p. 47–83.

[CIT0019] Irvin NA , Pierce C, Hoddle MS. Evaluating the potential of flowering plants for enhancing predatory hoverflies (Syrphidae) for biological control of Diaphorina citri (Liviidae) in California. Biological Control. 2021:157:104574.

[CIT0020] Khachatryan H , Rihn A, Behe B, Hall C, Campbell B, Dennis J, Yue C. Visual attention, buying impulsiveness, and consumer behavior. Market Lett 2018:29:23–35.

[CIT0021] Klecka J , Hadrava J, Biella P, Akter A. Flower visitation by hoverflies (Diptera: Syrphidae) in a temperate plant-pollinator network. PeerJ. 2018:6:e6025. 10.7717/peerj.6025 30533311 PMC6282941

[CIT0022] Kuehl RO. Design of experiments: statistical principles of research design and analysis. Pacific Grove, CA: Duxbury Press; 2000.

[CIT0023] Mach BM , Potter DA. Quantifying bee assemblages and attractiveness of flowering woody landscape plants for urban pollinator conservation. PLoS One. 2018:13(12):e0208428. 10.1371/journal.pone.0208428 30586408 PMC6306157

[CIT0024] Mader E , Shepard M, Vaughan M, Black SF, LeBuhn G. Attracting native pollinators: protecting North America’s bees and butterflies: the xerces society guide. North Adams, MA: Storey Publishing; 2011.

[CIT0025] Mallinger RE , Prasifka JR. Bee visitation rates to cultivated sunflowers increase with the amount and accessibility of nectar sugars. J Appl Entom. 2017:141(7):561–573.

[CIT0026] National Wildlife Federation. Enhancing conservation for native pollinators. Enhancing Conservation for Native Pollinators. Reston, Virginia, US: National Wildlife Federation (nwf.org); 2022.

[CIT0027] Ollerton J , Erenler H, Edwards M, Robin C. Extinctions of aculeate pollinators in Britain and the role of large-scale agricultural changes. Science. 2014:346:1360–1362. 25504719 10.1126/science.1257259

[CIT0028] Potts SG , Biesmeijer JC, Kremen C, Neumann P, Schwieger O, Kunin WE. Global pollinator declines: trends, impacts, and drivers. Trends Ecol Evol. 2010:25:345–353. 20188434 10.1016/j.tree.2010.01.007

[CIT0029] R Core Team. R: a language and environment for statistical computing. Vienna, Austria: R Foundation for Statistical Computing; 2021. https://www.R-project.org/.

[CIT0030] Rollings R , Goulson D. Quantifying the attractiveness of garden flowers for pollinators. J Insect Conserv. 2019:23(5–6):803–817. 10.1007/s10841-019-00177-3

[CIT0031] Silva EM , Dean BB. Effect of nectar composition and nectar concentration on honey bee (Hymenoptera: Apidae) visitations to hybrid onion flowers. J Econ Entomol. 2000:93(4):1216–1221. 10.1603/0022-0493-93.4.1216 10985033

[CIT0032] Skevington JH , Locke MM, Young AD, Moran K, Crins WJ, Marshall SA. Field guide to the flower flies of Northeastern North America. Princeton, NJ: Princeton University Press; 2019.

[CIT0033] Somme L , Vanderplanck M, Michez D, Lombaerde I, Moerman R, Wathelet B, Wattiez R, Lognay G, Jacquemart A. Pollen and nectar quality drive the major and minor floral choices of bumble bees. Apidologie. 2015:46:92–106.

[CIT0034] Theodorou P , Radzeviciute R, Lentendu G, Kahnt B, Husemann M, Bleidorn C, Settele J, Schweiger O, Grosse I, Wubet T, et al. Urban areas as hotspots for bees and pollination but not a panacea for all insects. Nat Commun. 2020:11(1):576. 10.1038/s41467-020-14496-6 31996690 PMC6989530

[CIT0035] Thomson D. Competitive interactions between the invasive European honey bee and native bumble bees. Ecology. 2004:85(458):458–470. 10.1890/02-0626

[CIT0036] (EPA) U.S. Environmental Protection Agency. Pollinator protection at EPA. Washington, DC: EPA; 2021.

[CIT0037] USDA-NASS. U.S. Department of Agriculture - National Agricultural Statistics Service. 2022. Honey bee colonies. Washington, DC: USDA; 2022a.

[CIT0038] USDA-NASS. U.S. Department of Agriculture - National Agricultural Statistics Service. 2022. Washington, DC: USA sales of annual bedding plants and herbaceous perennials. USDA/NASS QuickStats Ad-hoc Query Tool; 2022b.

[CIT0039] Van Rijn PCJ , Wäckers FL. Nectar accessibility determines fitness, flower choice and abundance of hoverflies that provide natural pest control. J Appl Ecol. 2016:53(3):925–933. 10.1111/1365-2664.12605

[CIT0040] White AJ , Wratten SD, Berry NA, Weigmann U. Habitat manipulation to enhance biological control of Brassica pests by hover flies (Diptera: Syrphidae). J Econ Entomo. 1995:88(5):1171–1176.

[CIT0041] Whitman C , Padhye S, Runkle ES. A high daily light integral can influence photoperiodic flowering responses in long day herbaceous ornamentals. Scientia Horticulturae, 2022:295:110897.

[CIT0042] Wignall VR , Alton K, Ratnieks FLW. Garden centre customer attitudes to pollinators and pollinator-friendly planting. PeerJ. 2019:7:e7088. 10.7717/peerj.7088 31211021 PMC6557251

[CIT0043] Wilde HD , Gandhi KJ, Colson G. State of the science and challenges of breeding landscape plants with ecological function. Hortic Res. 2015:2:14069. 10.1038/hortres.2014.69 26504560 PMC4596282

[CIT0044] Wojcik VA , Morandin LA, Adams LA, Rourke KE. Floral resource competition between honey bees and wild bees: is there clear evidence and can we guide management and conservation?. Environ Entomol. 2018:47(822):833. 10.1093/ee/nvy077 29873687

[CIT0045] Wollaeger H , Getter KL, Behe BK. Consumer preferences for traditional, neonicotinoid-free, bee-friendly, or biological control pest management practices on floriculture crops. HortScience 2015:50:721–732.

[CIT0046] Yeargan KV , Colvin SM. Butterfly feeding preferences for four Zinnia cultivars. J Environ Hortic. 2009:27(1):37–41. 10.24266/0738-2898-27.1.37

